# Vulnerability Assessment of Buildings due to Land Subsidence Using InSAR Data in the Ancient Historical City of Pistoia (Italy)

**DOI:** 10.3390/s20102749

**Published:** 2020-05-12

**Authors:** Pablo Ezquerro, Matteo Del Soldato, Lorenzo Solari, Roberto Tomás, Federico Raspini, Mattia Ceccatelli, José Antonio Fernández-Merodo, Nicola Casagli, Gerardo Herrera

**Affiliations:** 1 Geohazards InSAR Laboratory and Modeling Group (InSARlab), Geoscience Research Department, Geological Survey of Spain (IGME), Alenza 1, 28003 Madrid, Spain; p.ezquerro@igme.es (P.E.); jose.fernandez@igme.es (J.A.F.-M.); g.herrera@igme.es (G.H.); 2 Spanish Working Group on Ground Subsidence (SUBTER), UNESCO, 03690 Alicante, Spain; 3 Escuela Técnica Superior de Ingenieros de Caminos, Canales y Puertos, Universidad Politécnica de Madrid, 28040 Madrid, Spain; 4 Department of Earth Sciences, University of Firenze, Via Giorgio La Pira 4, 50121 Firenze, Italy; matteo.delsoldato@unifi.it (M.D.S.); federico.raspini@unifi.it (F.R.); mattia.ceccatelli@unifi.it (M.C.); nicola.casagli@unifi.it (N.C.); 5 Centre Tecnològic de Telecomunicacions de Catalunya (CTTC), Division of Geomatics, Avenida Gauss, 7 08860 Castelldefels, Spain; lorenzo.solari@cttc.cat; 6 Departamento de Ingeniería Civil, Escuela Politécnica Superior, Universidad de Alicante, P.O. Box 99, 03080 Alicante, Spain; 7 EuroGeoSurveys: Earth Observation and Geohazards Expert Group (EOEG), Rue Joseph II 36-38, 1000 Brussels, Belgium

**Keywords:** Sentinel-1, COSMO-SkyMed, Pistoia, land subsidence, vulnerability, fragility curves

## Abstract

The launch of the medium resolution Synthetic Aperture Radar (SAR) Sentinel-1 constellation in 2014 has allowed public and private organizations to introduce SAR interferometry (InSAR) products as a valuable option in their monitoring systems. The massive stacks of displacement data resulting from the processing of large C-B and radar images can be used to highlight temporal and spatial deformation anomalies, and their detailed analysis and postprocessing to generate operative products for final users. In this work, the wide-area mapping capability of Sentinel-1 was used in synergy with the COSMO-SkyMed high resolution SAR data to characterize ground subsidence affecting the urban fabric of the city of Pistoia (Tuscany Region, central Italy). Line of sight velocities were decomposed on vertical and E–W components, observing slight horizontal movements towards the center of the subsidence area. Vertical displacements and damage field surveys allowed for the calculation of the probability of damage depending on the displacement velocity by means of fragility curves. Finally, these data were translated to damage probability and potential loss maps. These products are useful for urban planning and geohazard management, focusing on the identification of the most hazardous areas on which to concentrate efforts and resources.

## 1. Introduction

Land subsidence is referred to as a slow sinking of the ground surface due to natural causes or human activities [1]. Even if it is described as a moderate and gradual geological process rarely generating casualties, land subsidence can be responsible for important economic losses in urban areas [2]. This issue reaches a new impact level in a climate change context, characterized by severe droughts and sea level rise [3,4]. On the one hand, natural land subsidence is usually related to geological processes like volcanism, peat oxidation, isostatic adjustments or tectonic processes [5,6]. On the other hand, anthropogenic land subsidence is caused by underground activities such as water withdrawal, oil and gas extraction, mining or tunneling and overloading of compressible sediments [7,8,9,10,11,12]

Until the deployment of global navigation satellite system (GNSS) satellite constellations, land subsidence monitoring was carried out using leveling techniques [13,14]. New global positioning systems have improved the flexibility of monitoring networks, allowing the direct measurement of 3D deformations [15], alone or combined with satellite data [16,17,18]. The launch of synthetic aperture radar (SAR) satellites, the first in 1992 (ERS 1/2), provided a new way to monitor land subsidence over wide areas [19]. Still underused for urban and land planning, SAR interferometry (InSAR) data have become the powerhouse tool of land subsidence studies benefiting from measurement density, accuracy, revisiting time and availability of historical datasets [15]. Recently, Raspini et al. [20] designed and implemented the first monitoring system based on radar data over the Tuscany Region, showing how SAR data can be successfully employed as geohazard management tools. The service is still ongoing and fully operational.

Tuscany is affected by land subsidence related to different causes, such as water overexploitation, extraction of deep geothermal fluids and overloading, with some relevant consequences on structures. One of the most important and well-known land subsidence areas in Tuscany is the Firenze–Prato–Pistoia basin where, since the early 1990s, land subsidence has been triggered by intense groundwater withdrawal for agriculture, tree nursery activities and industry in the zone between the municipalities of Prato and Pistoia. This phenomenon and its effects are well described in some of the first applications of the InSAR technique for land subsidence detection published in the scientific literature [21,22]. Additionally, as well as the expected land subsidence bowl in the center of the basin, a new deformation area was detected using the ERS 1/2 satellite monitoring system set up in the Tuscany Region. According to ENVISAT satellite SAR data, this new deformation bowl remained stable during the 2002–2010 period [23]. However, since 2014 this area has shown a subsiding trend by Sentinel-1 (S-1) data [24], located within the historical city center of Pistoia, where vulnerable Renaissance cultural heritage buildings are located. It is worth mentioning that active and older ground deformation evidence was already collected in the 1960–1970 decade by Fondelli [25] and Fancelli et al. [26] in this area.

Human, social and economic consequences of geological risks led to the development of numerous methodologies to calculate the vulnerability of structures and infrastructure, dealing with their effects. Since seismic, volcanic and flood vulnerability has been widely studied, geological risks associated to local or slow processes are still underdeveloped. Recent studies focused on landslides and rockfalls, developing methodologies that generate useful products for local authorities derived from remote sensing data [12,27,28,29,30,31]. Land subsidence is characterized by slow displacements that usually cover large areas and involve agricultural and economic activities as well as buildings, cultural heritages and linear infrastructures, generating important economic losses. In this work, the fragility curves methodology, proved to be valuable for different geological risks [32,33,34], has been used to calculate the probability of damage and estimate the potential damage (or loss), improving the knowledge and response of society to subsidence-related problems.

## 2. Study Area

### 2.1. Geological Setting

**Figure 1:**
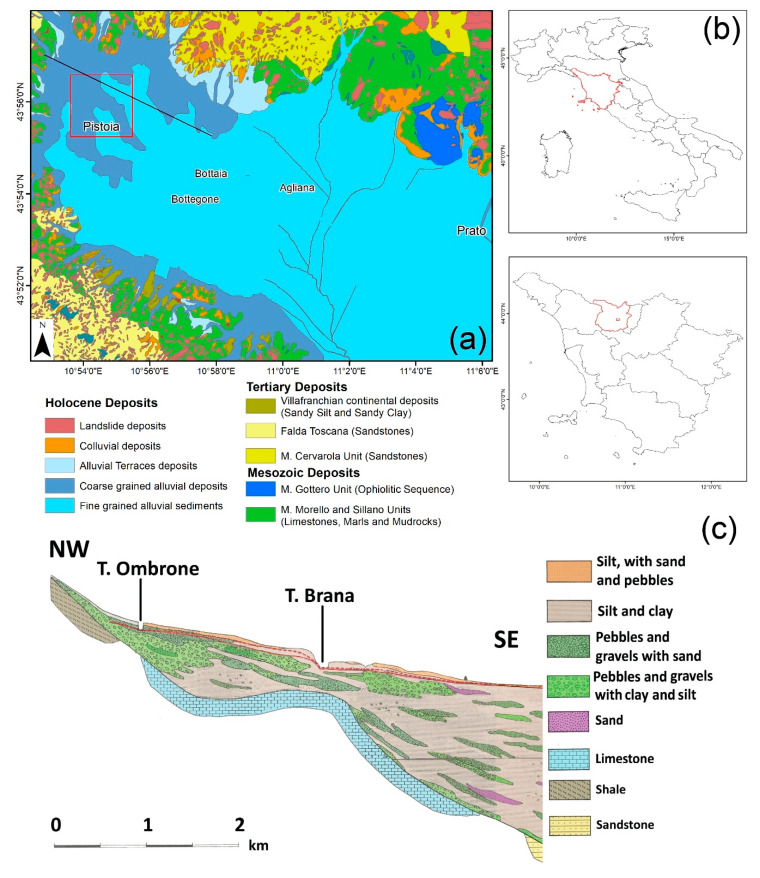
Geographical and geological context of the area of interest. Localization of the city of Pistoia: (**a**) geological map at 1:50,000 scale of the area of interest (**b**) (modified after Puccinelli et al. [38]). The red square shows the area of interest and the black line represents the trace of the geological section reported in (**c**) that has been adapted from Capecchi et al. [35]. The red dashed line represents the natural groundwater level of the basin.

The area of interest corresponds to the urban portion of the city of Pistoia, the main center of the homonymous province located in northeastern Tuscany (central Italy, Figure 1a). It is a typical medieval city with a population of approximately 91,000 inhabitants which hosts important cultural heritage sites, such as the Duomo and the San Giovanni Baptistery, that attract a multitude of tourists every year. A network of underground channels (called “gore” in Italian) runs below the historical city center. The “gore” channels, built in the Medieval Age, were used for water supply and today constitute a tourist attraction. In addition to the historical buildings and the cultural heritage sites, Pistoia is famous for its plant and flower nursery activities that are mainly widespread in the southeastern area of the city, called Bottegone.

Pistoia is located within the Firenze–Prato–Pistoia (Fi–Po–Pt) plain, a 35 km wide and 100 km long intermontane sedimentary basin with an average altitude of 45 m a.s.l. [35]. The basin is bounded by a major structural border ideally connecting Pistoia to Florence, 50 km southeast, corresponding to a normal fault SW dipping. The activity of this fault, since the Lower Pliocene, created an asymmetric semi-graben basin with a maximum depth of the substratum reaching 600 m in its northeastern portion [35]. The substratum is made of metamorphic Ligurian and Tuscan units, outcropping along the boundaries of the basin [36]. The tectonic depression has been progressively filled, starting from the Upper Pliocene, by alluvial, lacustrine and fluvial deposits. In general, the bottom of the stratigraphic sequence is comprised of clay and silt with organic or gravel levels. On top of this clayey layer, the sequence follows the paleogeographic evolution of the basin, with thick gravel and sand fan delta deposits at the mouth of lateral streams, in addition to sand and silt levels deposited by paleo-rivers wandering along the paleo-plain [35]. The stratigraphical asset is quite complex and varies a lot depending on the relative position within the basin. Strong lateral variations are common, determining a highly variable hydrogeological context with multilayer aquifers with different hydraulic properties.

The city of Pistoia rises along the northern boundary of the Fi–Po–Pt basin, and the stratigraphic asset reflects its position within the basin. Basement rocks outcrop a few kilometers north of the city center, being both Ligurian and Tuscan Domain metamorphic units (Figure 1b). The thickness of the sedimentary sequence varies along the axis of the basin (Figure 1c) between 30 m in the northwestern part of the city, to more than 100 m in the southeastern portion of the urban area. As said before, the stratigraphic asset shows a high lateral variability: thick gravel and pebble levels in the northern portion of the area of interest, coinciding with the Ombrone River fan delta, and prevalent silt and clay terrains with sparse and thin gravel and sand layers (with thickness frequently lower than 10 m) in the southeastern portion of the city.

The fan delta sequence hosts a phreatic aquifer that is largely exploited, thanks to its high transmissivity and quality of the resource, for the Pistoia aqueduct supply and for agriculture in the surrounding areas of Pistoia. In this area, a pumping well can reach a specific discharge of 5 to 10 l/s for 1 m drop of the groundwater level (usually found 1 to 5 m below the surface). It has been estimated that 85% of the drinking water supplied by the aqueduct of the city is extracted by pumping wells, with a maximum discharge of 150 l/s during summer. This phreatic aquifer is barely drained within the historic city center, where no industrial activities are found and where only a few wells extract water for air condition systems or for other usages. The aquifer is partially recharged by the water of the Ombrone River (losing stream disconnected, according to the definition of Winter et al. [37]), flowing in the western part of the city. In the southeastern part of the city, where clay and silt are more abundant, the phreatic aquifer is limited and not exploited. In this area, the source of underground water consists of thin, confined aquifers hosted by gravel or sand layer lenses found at different depths with low lateral continuity (Figure 1c). These aquifers, characterized by quite long recharge times, are intensively exploited by the flower and plant nursery activities that are located along the axis of the valley, south of Pistoia.

### 2.2. Previous Investigations

Land subsidence along the Fi–Po–Pt basin is a well-known phenomenon. Colombo et al. [21] and Canuti et al. [22] analyzed ERS 1/2 data, spanning between 1992 and 2001, to derive the first deformation map of the basin. These authors highlighted the presence of two main land subsidence bowls: one coinciding with the city of Prato (20 km south of Pistoia), where subsidence rates reached 2.0 cm/yr, and another one in the Bottegone area, recording a sinking up to 2.5 cm/yr. Land subsidence in the Prato area was connected to textile manufacturing, exploiting the thick phreatic aquifer of the Bisenzio fan delta. These industrial activities, started in the early 1960s and spread over the southern portion of Prato, depressed the phreatic level by several meters, inducing long-term subsidence [22]. Land subsidence in southern Pistoia was related to plant and flower nurseries, the main economic sector of the territory. As stated before, subsidence caused by these activities is triggered by the exploitation of several confined aquifers present at different depths.

Rosi et al. [23] and Del Soldato et al. [24] updated the interferometric analysis over the Fi–Po–Pt basin using ENVISAT data between 2003 and 2010 and S-1 data from 2014 to 2018, respectively. In this period, ground lowering in the city of Prato drastically decreased or stopped because of the recession of the textile industry that led to a lower exploitation of the aquifer [23]. In the Pistoia area, ENVISAT data confirmed the presence of a large subsidence bowl characterized by maximum subsidence rates higher than 3.0 cm/yr.

**Figure 2:**
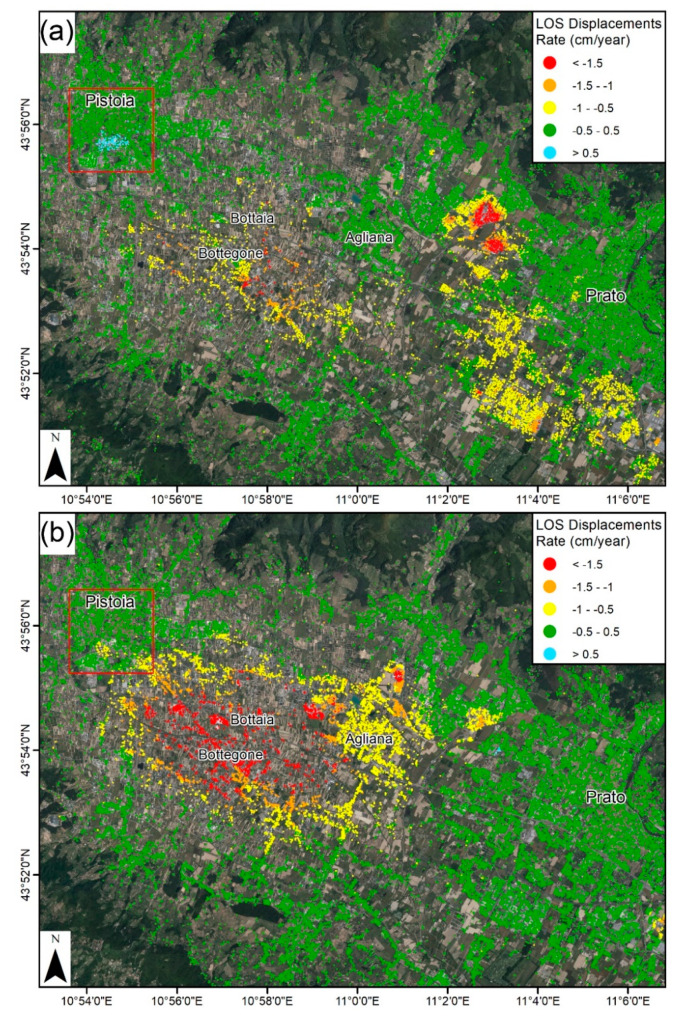
Land subsidence contours derived from ERS 1/2 (**a**) and ENVISAT (**b**) data, covering a time period spanning between 1992 and 2010. The red square represents the area of interest of this work where, starting from August 2016, a new lowering area has been discovered.

The results of these interferometric investigations in the Fi–Po–Pt basin are presented in Figure 2. ERS and ENVISAT datasets were processed using the PSInSAR algorithm [39], while the S-1 dataset results were obtained from the SqueeSAR algorithm [40], and analyzed by Rosi et al. [23] (ERS) and Del Soldato et al. [24] (ENVISAT and S-1). A stability threshold of 0.5 cm/yr, equal to double the standard deviation of the dataset, was set as the velocity lower limit for the delimitation of the subsidence bowl. The highest velocities were located between the Bottegone and Bottaia hamlets, ca. 8 km south of the city center. During the period covered by the ERS datasets (1992–2000), another important subsidence area was observed southeast of the Prato municipality. This active area was not identified in the subsequent performed ENVISAT analysis. In summary, in the 18-year investigated period, no evidence of land subsidence was recorded in the city center of Pistoia.

In the last few years, land subsidence has been recorded not only in the agricultural area of Pistoia but also in the city center, which was supposed to be stable [24]. According to the interferometric products used in this study, an anomalous variation of the spatial distribution of moving points was recorded, with a shape comparable to a subsidence bowl. It coincides with the historical city center, far from the already known southeastern subsiding area. The same area was already affected by an anomalous deformation event from 1964–1973 [25,26], confirmed by leveling measurements performed in the city center [25]. This leveling survey enabled the detection of up to 1.5 cm of subsidence for which no hypothesis about the triggering factors was proposed.

## 3. Materials and Methods

### 3.1. InSAR Processing and Analysis

S-1 C-band interferometric wide swath (IW) mode images, acquired in both orbits (86 in ascending and 93 in descending geometry), were processed by means of the SqueeSAR algorithm, developed by Ferretti et al. [40]. This algorithm can be considered the evolution of the PSInSAR (permanent scatterers interferometry) technique [39]. The idea behind SqueeSAR is to improve the way in which radar targets with stable coherence defined through the whole interferometric stack. PSInSAR is based on the definition of point-like targets named PS, corresponding to man-made objects or exposed rock. Exploiting the low temporal decorrelation and high level of backscattered signal of the targets, it is possible to derive reliable time series of deformation characterized by a high signal to noise ratio [41]. The SqueeSAR algorithm increases the number of radar target candidates by selecting partially coherent scatterers, named distributed scatterers (DS), corresponding to sets of pixels characterized by homogeneous amplitude values. These distributed targets correspond to bare soils, debris zones or uncultivated lands. The selection of the homogeneous pixels is performed through the non-parametric Kolmogorov–Smirnov (KS) test [42]. In brief, the KS test verifies, within a certain search window, if two or more pixels are statistically drawn from the same distribution function [39]. The SqueeSAR algorithm, by mixing the information retrieved from point-like and distributed targets, guarantees a high measurement points coverage not only in urban areas but also in peri-urban, rural or mountainous areas. Specific processing data of S-1 are summarized in Table 1.

S-1 data used in this work were derived from a specific project aimed to monitor the entire Tuscany Region territory by means of interferometric products [20], and were obtained by means of the SqueeSAR algorithm. The accuracy of the single radar measurement is around 0.5 cm and the geocoding error is equal to a few meters. Considering the number of images (>100) composing the 3-years-long interferometric stack, it is possible to estimate displacement rates with a precision of 0.1 cm/yr. More details on the processing chain, used dataset and characteristics of the operational service active in Tuscany can be found in Raspini et al. [20].

The anomalous deformation detected in Pistoia’s historical city center with the S-1-based monitoring system needed to be validated with external data. The absence of GNSS or leveling data in the area and the need for cross-comparison with high resolution radar data led to the request for COSMO-SkyMed (CSK) images [43]. Both datasets were processed with different techniques because S-1 data were processed as part of the semiautomatic Tuscany Region monitoring system and CSK data were requested from the Italian Space Agency (ASI) and processed as part of the presented analysis.

The CSK SAR data were acquired in both ascending and descending orbits and were processed using a different interferometric approach. The ascending stack was comprised of 60 Stripmap-Himages acquired from January 2015 to March 2018 with an almost regular temporal span of 16 days. The descending dataset consisted of 34 Stripmap-Himages acquired from February 2015 to December 2017 with a time span between 16 and 32 days and a maximum span of 96 days. Despite the availability of higher CSK temporal resolution (3–4 days maximum), the spatial distribution and temporal evolution of the studied land subsidence allowed selecting a longer image gap in order to reduce processing times while at the same time maintaining an appropriated level of temporal decorrelation.

CSK Stripmap (SM) mode images were processed using the coherence pixel technique (CPT), developed by the Remote Sensing Laboratory (RSLab) at Universitat Politecnica de Catalunya [44,45,46]. The CPT algorithm is based on the exploitation of spatial coherence, increasing the measurement points especially in rural areas through the use of distributed scatterers (DS). Two parts of the processing chain (PRISAR and SUBSOFT) are responsible for the coregistration, generation of interferograms, coherence maps and differential interferograms, and the estimation of linear velocities, time series and atmospheric filtering, respectively. Specific processing data of CSK are summarized in Table 1.

During the processing, a double minimization of temporal and spatial baselines was implemented in order to increase the deformation sensitivity and reduce the temporal decorrelation. This is crucial when analyzing high resolution X-band images over peri-urban areas. Descending dataset baselines were adapted to assure the connection and redundancy of the seven first images that presented extreme perpendicular baselines (around 50% longer in temporal and 100% larger in perpendicular than the ascending dataset). The selected multi-look (3 × 3) was adapted to improve the DS detection, preserving a good resolution in urban areas. In order to develop the non-linear calculation of the time series, a two-step atmospheric filter was applied. First, a spatial low pass filter, and then a high pass temporal filtering. The stability threshold of the results was settled on 0.5 cm/year, equal to 1.5 times the standard deviation values estimated for the points located in a stable area.

**Figure A1:**
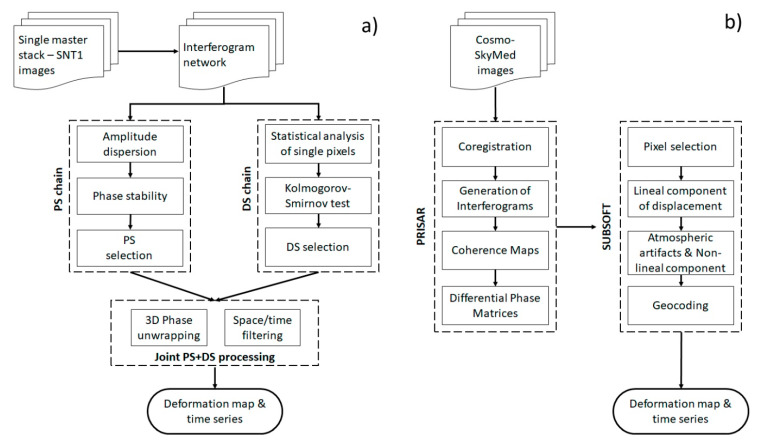
SqueeSAR (**a**) and coherence pixel technique (CPT) (**b**) processing workflows.

Although a summary of the algorithms used to process S-1 and CSK datasets used in this work is included in the subsections for the sake of completeness, a more detailed description can be consulted in [40] and [45], respectively, and the workflow of both processing chains is presented in Appendix A Figure A1.

Land subsidence is usually described as a main vertical movement, but this phenomenon can also generate horizontal displacements, sometimes difficult to detect [47,48]. Taking advantage of the two satellite acquisition geometries with both constellations, the east–west and up–down component of the deformation can be calculated and compared. The north–south component is assumed as negligible due to the quasi-polar orbits of SAR satellites [49]. S-1 and CSK line of sight (LoS) velocities were combined using a raster-based methodology [50,51]. InSAR velocities were interpolated with the inverse distance weighting (IDW) method [52,53], generating a coincident 50 m pixel raster, and then the up–down (V_UD) and east–west (V_EW) components of the velocity were calculated using the formulation from Notti et al. [51]. Since both geometries must overlap, the whole area of interest (2.5 × 2.5 km) was fully covered by the used datasets due to the large dimension of the used SAR images (300 × 300 km for S-1 and 30 × 30 km for CSK).

### 3.2. Field Surveys

Two field campaigns for damage level assessment of the edifices were carried out in the city of Pistoia. On-site surveys were performed on 26 June 2018 and 1 August 2018. During these field surveys, information on building characteristics as well as level and extent of damage, according to Del Soldato et al.’s [54] approach and scheme, were recorded for a total of 227 buildings. The 1:5000 official cadastral map of Pistoia [55] was used for the identification of the buildings. It is worth mentioning that: (a) these buildings were randomly selected trough several inspection tracks; (b) the inspection of the buildings was performed from the outer part of the buildings, since no access was possible for private properties; (c) most of the inventoried buildings were located in the historical downtown of Pistoia, presenting very similar structural characteristics (mainly masonry buildings); and (d) the inventoried damage was quite low, varying between G0 (no damage) and G2 (weak damage) according to the classification proposed by Del Soldato et al. [54].

For each surveyed building, the vertical displacements were derived from the decomposition of ascending and descending PS data available from S-1 and CSK datasets.

The combination of the level of damage of the recorded buildings and their associated vertical displacements (i.e., settlements) enabled us to build the fragility curves for each category of damage following the empirical methodology described in the next section.

### 3.3. Fragility Curves and Vulnerability Maps

In general, a fragility curve is a statistical tool representing the probability of reaching or exceeding a given damage state severity level ($D_{i}$) as a function of an engineering demand parameter, which usually defines the ground displacement [56]. Therefore, a fragility curve is a way to measure the vulnerability of structures in probabilistic terms.

Mathematically, the probability (P) of reaching a given damage ($D_{i}$) can be written according to the following equation [57]:

(1) $$P\left(Damage\geq D_{i}|\Delta \right)=\emptyset \left(\frac{1}{\beta }\cdot Ln\left(\frac{\Delta }{\bar{\Delta_{l}}}\right)\right)$$

where ∅ (∙) is the standardized cumulative normal distribution, and $\Delta_{l}$ and β are the median and the standard deviation, respectively, of the natural logarithm of the intensity parameter ∆.

In this work, the fragility curves were empirically built combining InSAR data and field data described in the previous section to evaluate the potential damage on buildings affected by land subsidence. The main advantage of this method is that it represents a realistic image of the real vulnerability of buildings since it is based on actual recorded damage and measured ground displacements [58].

Once the fragility curves have been calibrated using empirical data, they provide the probability of some level of damage for certain building displacement. Additionally, InSAR datasets allow the calculation of the vertical displacements that affect every building. Consequently, since InSAR data cover the whole city of Pistoia and provide information about the vertical displacement of all buildings, assuming a homogeneous structural typology, we can calculate the probability of damage (i.e., the vulnerability) within the whole city center of Pistoia. The results are represented using different maps for the deformations measured using each SAR sensor, and can be very useful to evaluate the extent of the city in which damage caused by land subsidence is expected. In this case, the observed damage was very low (G1 negligible or G2 weak) and most of the buildings exhibited a G1 degree. Consequently, we have grouped all damaged buildings into a unique group (including G1 plus G2), and thus two potential situations have been defined for the calculation of the fragility curves: no damage and damaged buildings.

Damage fragility curves were used to classify the velocities in five probability classes using the accumulated distribution. InSAR displacement rates were interpolated into a refined 5 m fishnet using the inverse distance weighting (IDW) method and reclassified using the previously calculated classes to generate the vulnerability maps.

**Figure A2:**
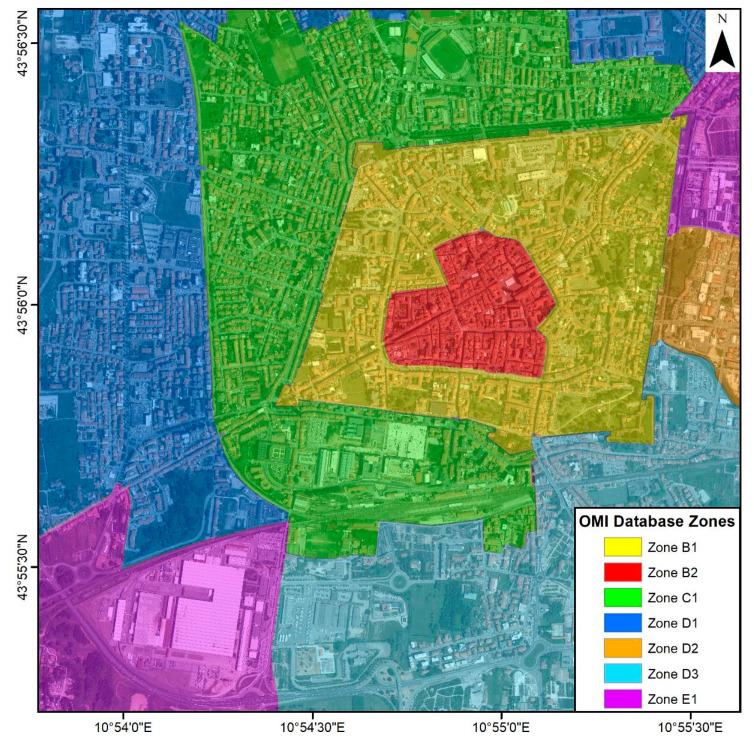
Osservatorio del Mercato Inmobiliario (OMI) database zones used to calculate the market value of the different building types (data from the OMI database—Italian Revenue Agency, 2019).

Introducing the economic value of buildings, it is possible to estimate the potential loss suffered by buildings in a quantitative way. We used the Italian government’s database (OMI database—Italian Revenue Agency [59]) which provides the market estimate of buildings depending on the real estate value of the district in which they are located. First, the mean value per square meter of different types of buildings (i.e., residential, offices, shops, industrial and warehouse) for every district of Pistoia was calculated. Then, the frequency of the different building types was calculated to estimate a mean market value per built square meter depending on the district (Table 2, Appendix A Figure A2).

Threatened assets can be estimated on each cadastral plot using the formulation described by Wiebe and Cox [60]:

(2) $$V_{a}=MV\cdot P$$

where $V_{a}$ is the vulnerable assets (Euros/m^2^), MV is the market value of each cadastral plot (Euros/m^2^) and P is the probability of damage (%) obtained from frequency curves.

A more realistic calculation of possible losses was obtained adapting the methodology from Goda and Song [61], using the market value instead of the replacement costs of that work.

(3) $$L=MV\cdot P\cdot R_{L}$$

where L is the losses (Euros/m^2^), MV is the market value of each cadastral plot (Euros/m^2^), P is the probability of damage (%) obtained from frequency curves and $R_{L}$ is the loss ratio, a percentage that represents the percentage of market value damaged depending on the damage level. Following the $R_{L}$ values described by Goda and Song [61], the damage level observed in Pistoia was similar to the minor damage of those works, assigning $R_{L}$ = 0.05.

## 4. Results

### 4.1. InSAR Processing and Analysis

**Figure 3:**
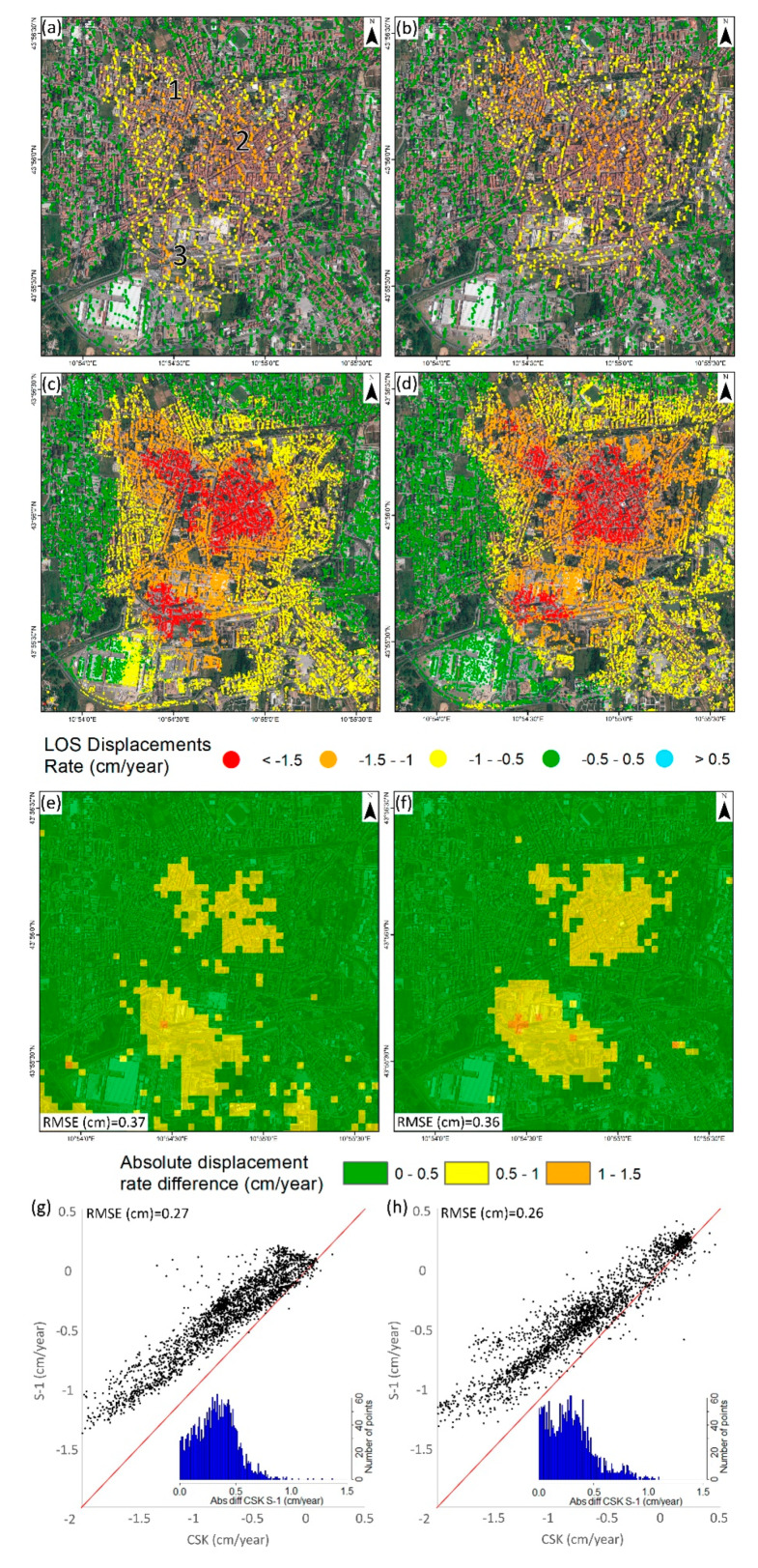
S-1 ascending (**a**) and descending (**b**) displacement velocities, CSK ascending (**c**) and descending (**d**) displacement velocities, spatial comparison of ascending (**e**) and descending (**f**) results and CSK-S-1 dispersion plots and histograms of ascending (**g**) and descending (**h**) geometries. Numbers in Figure 3a represent the area of the Figure 4 time series: 1 City NW, 2 City Center, 3 City SW.

The first set of deformation maps was obtained using ascending and descending S-1 data acquired in the framework of the continuous InSAR monitoring over Tuscany (Figure 3a,b). As previously highlighted in Section 2, subsidence detected in the SE portion of Pistoia has already been known since the early 1990s. S-1 data allowed the detection of a new subsidence bowl affecting the Pistoia historical city center, reaching a maximum rate of −1.4 cm/year in both the ascending and descending geometries. Another smaller land subsidence bowl with a higher rate (−1.7 cm/year) was detected in the SW area, near the river. The stability range (±0.5 cm/year) was selected using the standard deviation of the dataset.

Considering that most of the subsidence bowl is located in an urban area, CSK images were acquired and processed, improving the spatial density of PS measurements. CSK data allowed the validation of S-1 results considering the lack of external sources of ground deformation measurements. The high-resolution CSK data results showed maximum displacement rates of −2.3 cm/year in both geometries (Figure 3c,d). High displacement rates (−2.7 cm/year) were detected in the SW river area, as observed in the S-1 results. CSK results were classified using the same stable range as S-1 (±0.5 cm/year) to enable the comparison between both datasets. CSK displacements showed a wider subsiding area than S-1, and also with higher rates in the most affected areas. These results differ especially in the SE side of the city, where CSK data showed a subsidence area linking the historical subsidence area and the new one detected.

In order to carry out the spatial cross-validation of both satellites’ datasets, the results were interpolated into a 50 × 50 m raster fishnet using an IDW (Figure 3f). The absolute difference of the ascending and descending results was recorded approximatively under 0.5 cm/year (most restrictive stable range) over most of the studied area, and the root mean square error (RMSE) was below this threshold as well (0.37 and 0.36 cm/year, respectively). The concentration of values under 0.5 cm/year is clearly observed on the absolute difference histograms. Dispersion graphics (Figure 3g,h) reveal there exists a deviation of the data with respect to the identity line (RMSE of 0.27 cm/year in the ascending and 0.26 cm/year in the descending). This fact suggests that CSK velocities are consistently higher than S-1 in the analyzed area, although this fact must be confirmed by the time series.

**Figure 4:**
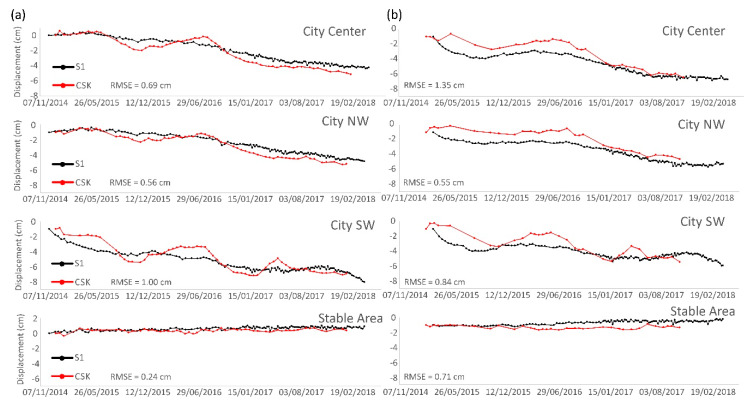
Ascending (**a**) and descending (**b**) time series over the main deformation areas. These points are located in Figure 3a. The stable area is outside of the main area of interest.

LOS velocities are useful to perform spatial analysis and to detect small scale trends, but time series allow further analysis. Three representative sectors of the city (Figure 3a) were selected to explain the temporal behavior of subsidence over the analyzed period (Figure 4). Despite the slight differences detected in velocities, the time series show good agreement. The RMSE calculated in coincident dates was below 1 and 1.35 cm in the ascending and descending (Figure 4). Since both series present similar behavior, the more variable results of CSK data increased the final error. The higher error of the descending dataset was related to the lower number of CSK images (34 with respect to 60 in the ascending) processed that generate a noisier result.

The good agreement between CSK and S-1 time series lead to a reinterpretation of the deviation of the velocities. In this case, the mean velocity calculation method is essential because it can substantially change the results. Since CPT calculates the linear regression slope as the mean velocity and CSK time series are more sensible, it resulted in higher mean velocities than S-1. The presented cross validation of the InSAR results indicates that the results have a good spatial and temporal agreement, but both velocities and time series must be compared to assure the results.

**Figure 5:**
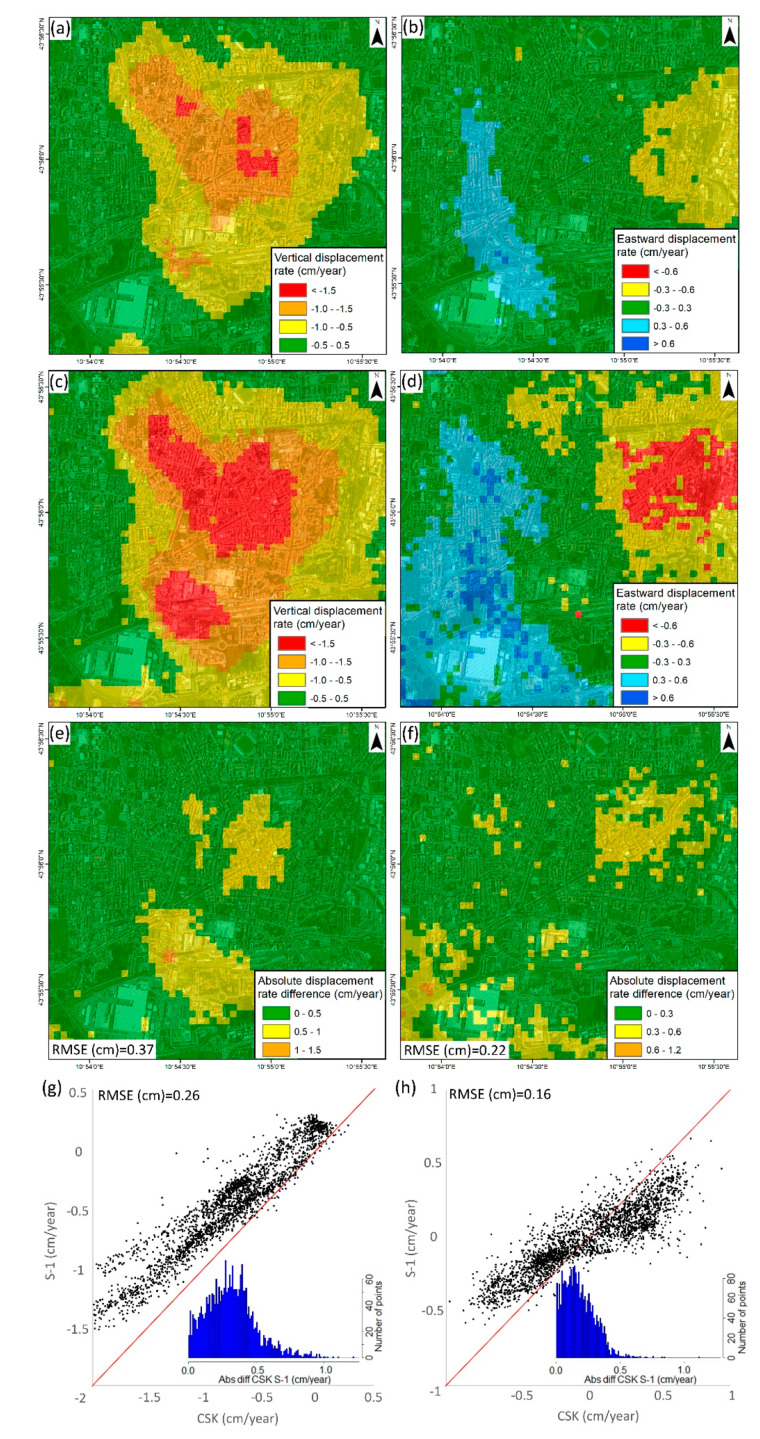
S-1 vertical (**a**) and EW (**b**) displacement velocities, CSK vertical (**c**) and EW (**d**) displacement velocities, and spatial comparison of vertical (**e**) and EW (**f**) results, and CSK-S-1 dispersion and histogram plots of vertical (**g**) and EW (**h**) geometries.

The slight differences in the displacement pattern detected between the ascending and descending suggest the possible existence of a horizontal component of motion. This kind of displacement is sometimes detected in subsidence areas because of lateral strains [62,63]. To detect them, the vertical and E–W components were separately analyzed (Figure 5). The vertical component of S-1 and CSK present similar patterns, with the main subsidence bowl coinciding with the city center and the secondary area (SW of the Ombrone River). The SE area shows greater subsidence in the CSK data, with a spatial coverage comparable to what was detected by ERS and ENVISAT data. Comparing S-1 and CSK vertical velocities, the same deviation trend (RMSE with respect to the identity line of 0.26 cm/year) observed in the analysis of the ascending/descending data was observed. The RMSE (0.37 cm/year) was below the stability range (±0.5 cm/year) and the histogram concentrated the differences under that value. For the horizontal component, the stability range was set as ±0.3 cm/year depending on the standard deviation value. Two slow but consistent areas moving towards the subsidence center, equal to 0.5 cm/year and 0.7 cm/year were detected for S-1 and CSK, respectively. Calculated absolute differences, RMSE (0.22 cm/year) and the histogram were below this value. As the E–W component is highly dependent on the spatial differences between both geometries, the dispersion plot shows a good agreement between CSK and S-1 with a low RMSE (0.16 cm/year).

### 4.2. Field Surveys Maps

**Figure 6:**
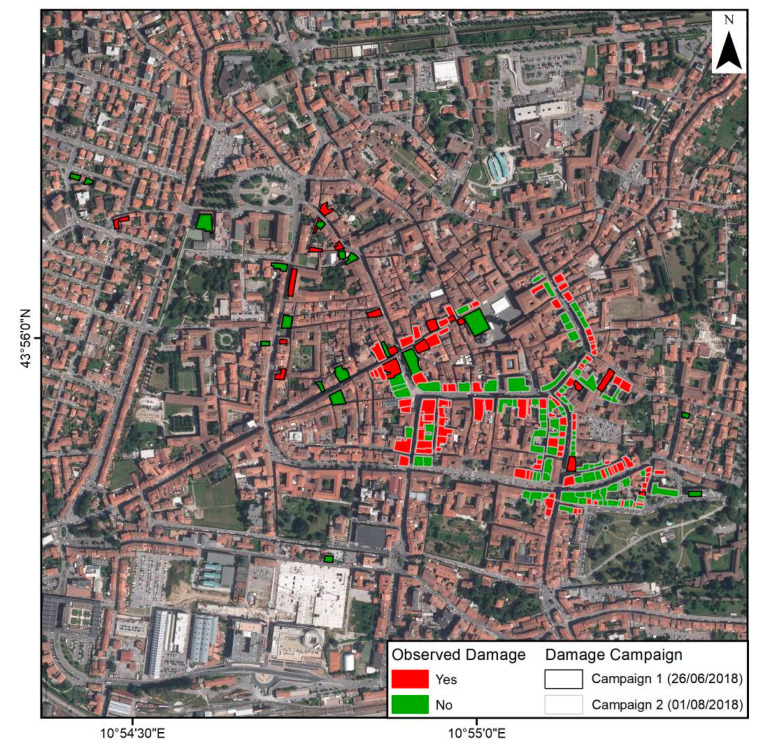
Results of the in-situ damage detection field campaigns.

Damage detection field campaigns, focused on the city center, resulted in 227 observed buildings (39 in the first campaign and 188 in the second one). The first campaign was designed as an initial approach to the problem, trying to cover all the affected area but not exhaustively investigating all the buildings along the path. The second campaign focused on a small section of the Pistoia city center, cataloguing the health condition of all the structures in that area. Although the first campaign database is scarce and randomly generated, it allowed covering a larger displacement variety, necessary to better define fragility curves. The adoption of a simple methodology to carry out a reliable quick damage assessment and low damage level detected (i.e., all buildings exhibited a G0 no damage to G2 weak damage) led us to classify the buildings as damaged or non-damaged (Figure 6) in order to improve the clarity of the results to an untrained final user. Forty-seven percent of the buildings do not exhibit any level of damage; meanwhile, 53% of them present different types of cracks and damage.

### 4.3. Fragility Curves and Vulnerability Maps

**Figure 7:**
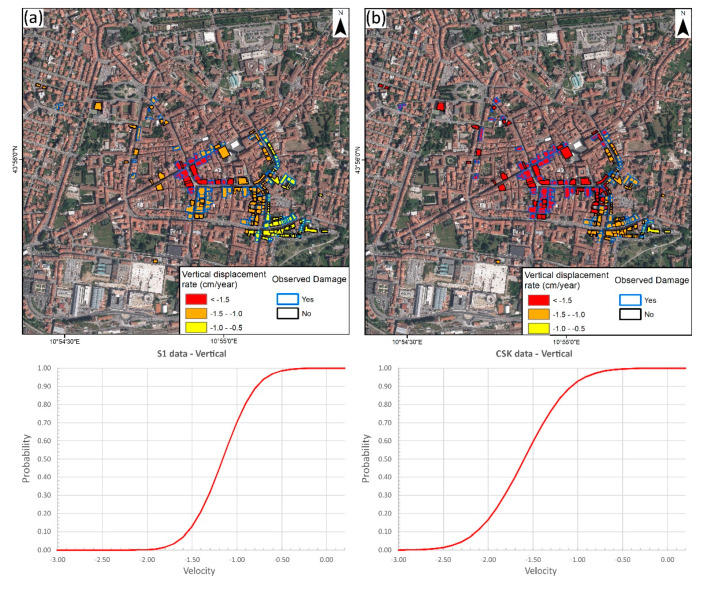
Mean vertical velocities of the inspected buildings in S-1 (**a**) and CSK (**b**), and fragility curves of the damaged buildings for them.

Using the two available datasets and the damage information, two fragility curves for masonry-type buildings, one for each satellite, were calculated (Figure 7). Using the vertical displacements, the S-1 damage curve ranges from 0% of damage probability at −0.4 cm/year to 100% at −2 cm/year, describing a steep curve. The CSK curve shows a similar distribution with values ranging from −0.5 (0%) to −2.6 cm/year. Observing the higher amplitude of CSK data, covering a higher displacement range, they better represent a smooth distributed phenomenon like land subsidence.

**Figure 8:**
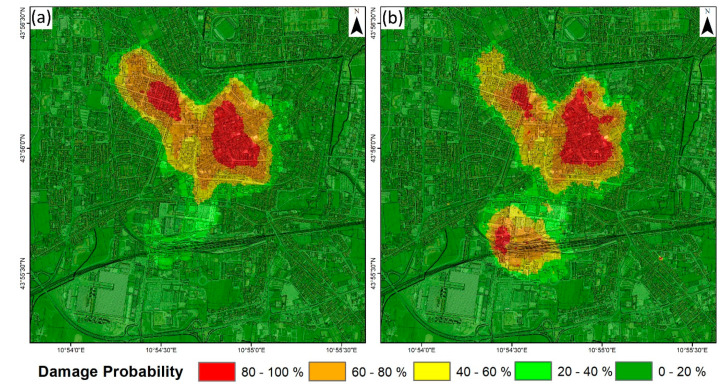
Damage probability spatial distribution calculated using the S-1 (**a**) and CSK (**b**) fragility curves.

Vulnerability maps from S-1 and CSK (Figure 8) present a major difference for the south part of the city center. Less steep CSK ascending fragility curves result in a smoother vulnerability map that better covers all the displacement velocities’ range. This solution is also the most conservative one, generating the largest vulnerable area (0.8 km^2^ above 40% of damage probability).

**Figure 9:**
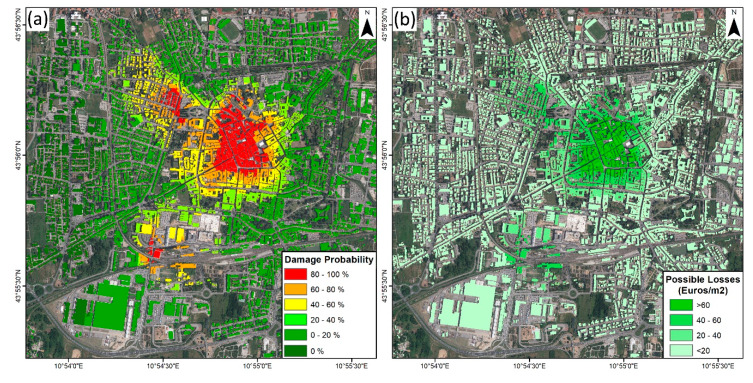
(**a**) Damage probability of each cadastral plot. (**b**) Vulnerable assets.

Using the cadastral information from Pistoia City, the probability of each building suffering damage due to the detected subsidence was calculated. It is worth noting that to perform this analysis, we assumed that the typology of the buildings across Pistoia was homogeneous and similar to that of the surveyed buildings from which fragility curves were defined. The mean velocity of each cadastral plot and the probability of damage was also evaluated (Figure 9). The results show that 4.3% of the buildings are within the high-vulnerability class (80–100% probability of damage). Additionally, 16.9% of the buildings are out of the vulnerable area, where damage probability due to the studied phenomenon is null. From 9257 cadastral plots with probability values between 0% and 80%, 86% of them are below 40% of damage probability. It is worth noting that in this case, most cultural heritage sites (e.g., Battistero di San Giovanni in Corte, Cattedrale di San Zeno, Palazzo Pretorio) and some critical structures (e.g., Pistoia train station) are in the high-vulnerability area, where economical value is way higher (train station) and even difficult to estimate (cultural heritage sites).

Vulnerable assets show a maximum potential loss of 1500 Euros/m^2^ in the city center, combining the effect of high probability of damage and market value. More than 652 buildings have an estimated loss of over 1000 Euros/m^2^, representing 5.5% of those analyzed. In addition, 64.8% show a residual cost below 100 Euros/m^2^, mainly derived from the continuous distribution of probability maps. Expected losses, calculated using the formulation of Goda and Song [61], were lower than potential ones due to the low damage level observed during the survey campaigns in the subsidence area. Maximum probable losses only reached 77 Euros/m^2^, and 42.4% of the buildings present had an expectable damage value of less than 1 Euro/m^2^.

## 5. Discussion

In this work, we evaluated the capabilities of large-scale processing based on S-1 data to detect and characterize new deformation areas. A first comparison of historical land subsidence in the Fi–Po–Pt basin, measured with ERS (1992–2000) and ENVISAT (2003–2010), with recent S-1 presented a similar pattern and decreasing maximum subsidence rates in the last period from 3.4 cm/year to 1.3 cm/year [24]. The comparison of S-1 data with high-resolution CSK images provided interesting results. Slightly higher rates of CSK than S-1 over the Bottegone area fitted better with the spatial extension of ENVISAT subsidence; meanwhile, maximum subsidence rates only reached 1.8 cm/year. The cross-validation was also successful in the newly detected subsiding areas in Pistoia’s city center, with spatial and temporal RMSE below stability ranges. In addition, two-dimensional decomposition of the movements was performed in the Pistoia city center, revealing the existence of horizontal displacements toward the center of the subsidence area. Those small but consistent horizontal displacements are for urban areas due to their influence in the structures’ stability. Going beyond the results validation, CSK data, focused on urban areas, allowed the improvement of the vulnerability analysis from a neighborhood (S-1) to cadastral plot scale (CSK).

The triggering mechanism of the detected land subsidence is still under investigation. Meanwhile, subsidence wide distribution (2.23 km^2^) and slow displacement velocity (below 3 cm/year) agrees with the behavior of historically detected subsidence in the basin, mainly related to groundwater changes. The lack of groundwater level data in the urban area prevented us from supporting the changes in the aquifer system as the triggering mechanism. A main hypothesis of those changes focuses on the anomalous or unknown groundwater overexploitation or the oversaturation and following subsidence of the area already described in 1960′s documents. The other considered hypothesis, soil consolidation, is local and usually associated to recently built structures, hardly supported in the part of the subsidence area that dates from the Middle Ages. Ongoing studies are obtaining hydrogeological data to improve the knowledge of the processes occurring in the area.

Field surveys carried out to generate the frequency curves and damage probability maps were designed by considering damage on facades and considering all the buildings as an only constructive typology. Validation of the damage probability was analyzed calculating the damaged/inspected buildings on each damage probability range (Table 3). Considering the number of inspected buildings and the validation methodology adopted, the frequency curves definition and validation was performed using the same data to maintain statistical consistency. Central ranges (20–80) showed a good performance, with values in the respective ranges. Extreme ranges (0–20 and 80–100) revealed an underestimation and overestimation of the damaged buildings, respectively. Taking into account the existence of buildings from the 12th century with masonry foundations to the 20th century with recent founding techniques, the simple approach used in this work could misestimate the damage in the most vulnerable areas or overestimate the subsidence effects over reinforced concrete buildings. Another possible explanation of the existence of buildings with lower damage than expected is the recent beginning of the land subsidence process. Studied subsidence began in early 2015 with a slight acceleration in the summer of 2016, but still slow subsidence rates (below 3 cm/year). These slow phenomena usually generate damage in the long term and the damage detected in this work is still emerging. Last, the surveys only inspected the outer side of the buildings, hiding the possible existence of damage in their inner part. In addition, recent restoration of the buildings could mask the potential damage, as observed in some buildings surveyed in the study area.

Vulnerable assets and potential losses were calculated using information from the OMI database (Osservatorio del Mercato Inmobiliario). This database subdivides each municipality into subzones where the Italian government collects real market value information every year. Scientific users also consider the OMI database as a reliable source of information, exploiting it in other geohazards like landslides [31,64]. In this work, we were for the first time using this information, joint with fragility curves, to estimate the possible impact of land subsidence in an urban area. Use of detailed data from cadastral units (floors, economic use, area) would allow a better calculation of the threatened assets, but this is the most accurate information currently available at a regional scale with a reasonable time investment. It should also be noted that, as a medieval city, Pistoia has several invaluable cultural heritage buildings that could be damaged.

Considering that current damages only reach negligible (G1) or weak (G2) levels, two approaches were followed in order to evaluate the affection of land subsidence. On the one hand, we used Wiebe and Cox’s [60] formulation to evaluate the vulnerable assets if land subsidence continues and causes severe damage on buildings that imply the total loss of value. On the other hand, small cracks and fissures currently observed do not threaten the structural safety and do not imply a loss of the total cost of the affected buildings, so a more reasonable estimation of current losses using the correction factors from Goda and Song [61]. The final results suggest that expected losses at this moment can be repaired spending a few hundred euros. Regular damage campaigns (e.g., once a year), extended to a larger portion of the city center and including the inner parts of the buildings, will guarantee the response of the buildings to better follow the subsidence trend variations. This will certainly increase the accuracy of the fragility curves, enlarging the training dataset and improving the estimation of the potential damage, and thus of the potential loss. Such periodical surveys should be accompanied by a regular processing plan for the satellite data, as the Tuscany Region is performing at the moment. Further improvements will consist of the installation of crackmeters on the buildings showing the most severe damage level. This mixed ground/satellite-based monitoring system could also be completed by periodical topographical campaigns.

Fragility curves are a common resource for damage prediction and risk management in seismic and tsunami threatened regions. These curves are calculated in areas with similar geological framework and building typologies, and then used to predict the impact of the hazard on the selected area [60]. In this work, we have empirically calculated fragility curves from field observations. They are useful tools for urban planners since they enable the evaluation of the spatial probability of damage due to land subsidence processes. Regarding this topic, the Tuscany Region government released on February 2019 a regional procedure for geohazard management (Resolution n° 224 of 25/02/2019), which includes InSAR-based monitoring data for preliminary risk assessment, deriving actions that should be taken depending on the risk level [65]. Following the same aim, the results obtained in this work would be useful for urban planners to predict those areas of the city in which damage could develop if land subsidence continues or even if new areas are affected by land subsidence.

## 6. Conclusions

This paper proposed a complete methodology, from automatic detection to building damage probability, for risk assessment in land subsidence areas. We generated a multi-scale observation system based on S-1, which is valuable to scan wide areas and to spot unstable areas, and CSK for detailed analysis on relevant targets. Land subsidence detected using an automatic tool based on S-1 remote sensing data have been validated using high resolution CSK images. Ascending and descending datasets allowed the decomposition of displacements in vertical and E–W directions and detected slow but continual horizontal displacements of 0.5 cm/year towards the city center.

The developed field survey for the inventory of damage permitted us to generate fragility curves from S-1 and CSK data. Using the more sensitive CSK data, damage probability was calculated for each cadastral plot of the Pistoia city center, resulting in 4.3% of the buildings in the 80–100% damage probability range and 22.9% over 40%. The final damage probability predicted for individual cadastral plots established a first impact scenario that must be reevaluated and validated with updated displacement and damage campaigns results. Lastly, an estimation of the subsidence-related threatened assets was calculated to highlight the importance of the problem, with an average value of 205 Euros per built squared meter. The estimation of affected assets used a simple methodology to calculate the potential impact of land subsidence. Final estimation of the possible losses, applying a 5% loss ratio due to the low level of the damage detected, reached a maximum of 77 Euros/m^2^ with 82% of the area of interest under 20 Euros/m^2^.

This case study has allowed analyzing a new subsidence area while it happens. Usually this kind of analysis is performed when serious problems are detected (big cracks, infrastructure damage, etc.), various years after their beginning. The analysis performed achieved the creation of valuable and easily understandable information and an estimation about the spatial extension, gravity and probability of the studied phenomenon useful for urban planners.

## Figures and Tables

**Table 1:** COSMO-SkyMed (CSK) and Sentinel-1 (S-1) processing parameters.

Satellite	CSK Ascending	CSK Descending	S-1 Ascending	S-1 Descending
Number of images	60	34	136	128
First image	7/1/2015	22/2/2015	12/12/2014	22/3/2015
Last image	4/3/2018	26/11/2017	13/5/2018	17/5/2018
Number of interferograms	287	141	-	-
Processed area (km^2^)	112	69	32,529.6	29,173.5
Max. temporal baseline (days)	288	443	-	-
Max. spatial baseline (m)	598	1146	-	-
Multilook (Az × Rg)	3 × 3	3 × 3	-	-
Number of DS	95,485	84,279	501,201	365,553
PS + DS Density (PS + DS/km^2^)	852.5	1221.4	15.4	12.5

**Table 2:** Value and frequency of the different economical uses by district.

	Building Type										
District	Houses		Industry		Office		Shops		Warehouse		Final Value
	€/m^2^	%	€/m^2^	%	€/m^2^	%	€/m^2^	%	€/m^2^	%	€/m^2^
B1	1525	26.5	800	13.7	1275	22.2	1450	24.8	750	12.8	1252.4
B2	1810	31.3	0	0.0	1575	27.0	1675	28.8	750	12.9	1571.2
C1	1510	25.1	800	13.6	1400	23.8	1450	24.7	750	12.8	1275.3
D1	1590	26.7	825	14.2	1325	22.8	1375	23.7	775	12.5	1267.8
D2	1425	23.0	760	14.4	1400	23.0	1575	25.5	875	14.0	1284.8
D3	1440	24.8	775	14.3	1400	24.3	1450	24.3	725	12.2	1250.2
E1	1460	25.2	635	13.9	1375	23.9	1425	25.7	625	11.3	1221.5

**Table 3:** Validation results of the damage probability maps.

Damage Probability Range (%)	(0–20)	(20–40)	(40–60)	(60–80)	(80–100)
No Not Dmg. Buildings	30	31	22	10	14
No Dmg. Buildings	32	20	20	18	30
Percentage of Damaged (%)	52	39	48	64	68
